# High-speed radiography of laser-induced shock wave generation in a mock explosive

**DOI:** 10.1038/s41598-026-49885-2

**Published:** 2026-06-09

**Authors:** H. Lorenté, A. Mouskeftaras, A. Sollier, B. Lukic, A. Rack, O. Uteza, L. Jumpertz

**Affiliations:** 1 French-German Research Institute of Saint-Louis (ISL), 5 rue du Général Cassagnou, Saint-Louis, 68300 France; 2 Aix-Marseille University, CNRS, LP3 UMR 7341, Campus de Luminy, Case 917, CEDEX 09, Marseille, 13288 France; 3 CEA, DAM, DIF, Arpajon, 91297 France; 4 Laboratoire Matière en Conditions Extrêmes, Université Paris-Saclay, CEA, Bruyères-le-Châtel, 91680 France; 5 ESRF-The European Synchrotron, 71 Avenue des Martyrs, Grenoble, 38000 France

**Keywords:** Nanosecond laser, Time-resolved radiography, Shock waves, Sucrose, Materials science, Optics and photonics, Physics

## Abstract

Short-pulse laser-induced shock waves can produce high-pressure, short-duration mechanical loading in granular energetic media. To quantify this phenomenon, we performed experiments in a standard mock explosive -compressed sucrose- avoiding the handling constraints of explosives. Because the medium is granular and optically opaque, we employed time-resolved synchrotron X-ray imaging to directly observe shock-induced structural evolution. A green nanosecond laser pulse serves as the shock driver. The generation of a shock wave is confirmed by ex-situ tomographic analysis, which also enables quantitative interpretation of the time-resolved data to determine the shock pressure (~ 3.6 GPa) and propagation velocity (~ Mach 4). Furthermore, doping with aluminum nanoparticles enhances laser absorption and increases the resulting shock pressure. These results demonstrate the potential of high-power laser pulses for initiation of detonation in secondary explosives and provide a pathway toward faster, optically-triggered detonator concepts.

## Introduction

Conventional pyrotechnic detonators—widely used in aerospace, defense and industry—are electrically initiated and therefore vulnerable to electromagnetic interference^1^. Opto-pyrotechnic detonators were developed to mitigate this risk by enabling remote optical triggering and improved safety. Existing optical approaches include laser-heated thermal igniters^2,3^, laser-driven flyer/slapper systems^4,5^, and direct laser–material interaction strategies^6,7^. Thermal and flyer methods are robust but either slow (thermal heating) or mechanically complex (multi-layer flyers), motivating interest in direct optical initiation that exploits the ultrafast energy deposition of lasers to produce shock-to-detonation transitions^8^. Opto-pyrotechnic detonators offer the additional advantage to contain only highly-pressed secondary explosives such as hexogen (RDX), which present reduced sensitivity compared to primary explosives but require strong, short-rise shocks to initiate detonation. Nanosecond laser pulses are attractive for generating localized, high-amplitude shock waves^9^: in wide-bandgap energetic materials, sub-bandgap light is first absorbed via defect-assisted primary ionization, followed by avalanche ionization and inverse Bremsstrahlung heating of the nascent plasma^10^. In the nanosecond regime the plasma continues to absorb during the pulse, driving a rapid pressure rise at the surface and launching a shock into the material^11^.

While laser–granular interactions and laser-driven modification of weakly absorbing powders have been reported, detailed studies of laser-induced shock propagation in weakly absorbing granular materials that mimic high explosives are scarce. Impact-based shock studies^12^ provide valuable insight into compaction and stress transmission but differ fundamentally from laser-generated shocks in rise time and front morphology. These differences complicate the transfer of impact-based knowledge to laser shock-driven initiation.

Time-resolved optical diagnostics^13,14^ are often hampered in highly scattering granular samples while rear face velocimetry^15^ can measure the surface velocity but do not provide detailed spatial information. Time-resolved X-ray imaging^16–18^—available at high-brightness facilities such as the ID19 beamline at the European Synchrotron Research Facility (ESRF)^19^—overcomes this limitation by providing high spatiotemporal fidelity in transmission and phase contrast imaging, and it has proven effective for in-situ studies of laser-driven shocks in low-density materials^20^. The mechanisms of micrometer-scale hot-spot formation governing the early stages of shock initiation in energetics materials have recently been characterized through time-resolved X-ray imaging of shock-induced pore collapse in HMX single crystals^21^.

Here, we combine synchrotron X-ray dynamic imaging and high-resolution post-mortem tomography to investigate nanosecond laser-induced shock generation and propagation in a mock explosive, compressed sucrose. Our goal is to characterize shock dynamics and assess conditions relevant to the optical initiation of secondary energetic materials.

## Methods

### Selection, fabrication, and characterization of mock explosives

In this work, mock explosive samples are used instead of high explosives for safety reasons. They are granular materials, whose optical properties and shock response are representative of those of the explosive. Sucrose ($\:{C}_{12}{H}_{22}{O}_{11}$) is used, as it is a standard mock explosive^22^ easily available, whose shock parameters are well-known, enabling quantitative characterization. The compared refractive index, linear absorption coefficient and density of sucrose and RDX are indicated in Table 1.

**Table 1:** Properties of sucrose and RDX at$\:\lambda\:=532\:nm$.

Material	Sucrose	RDX
Refractive index	1.55^23^	1.60^24^
Linear absorption coefficient$\:\left(\mathrm{c}{\mathrm{m}}^{-1}\right)$	0.025^25^	0.050^﻿26^
Density$\:(\mathrm{g}/\mathrm{c}{\mathrm{m}}^{3})$	1.58	1.80^27^

**Fig. 1:**
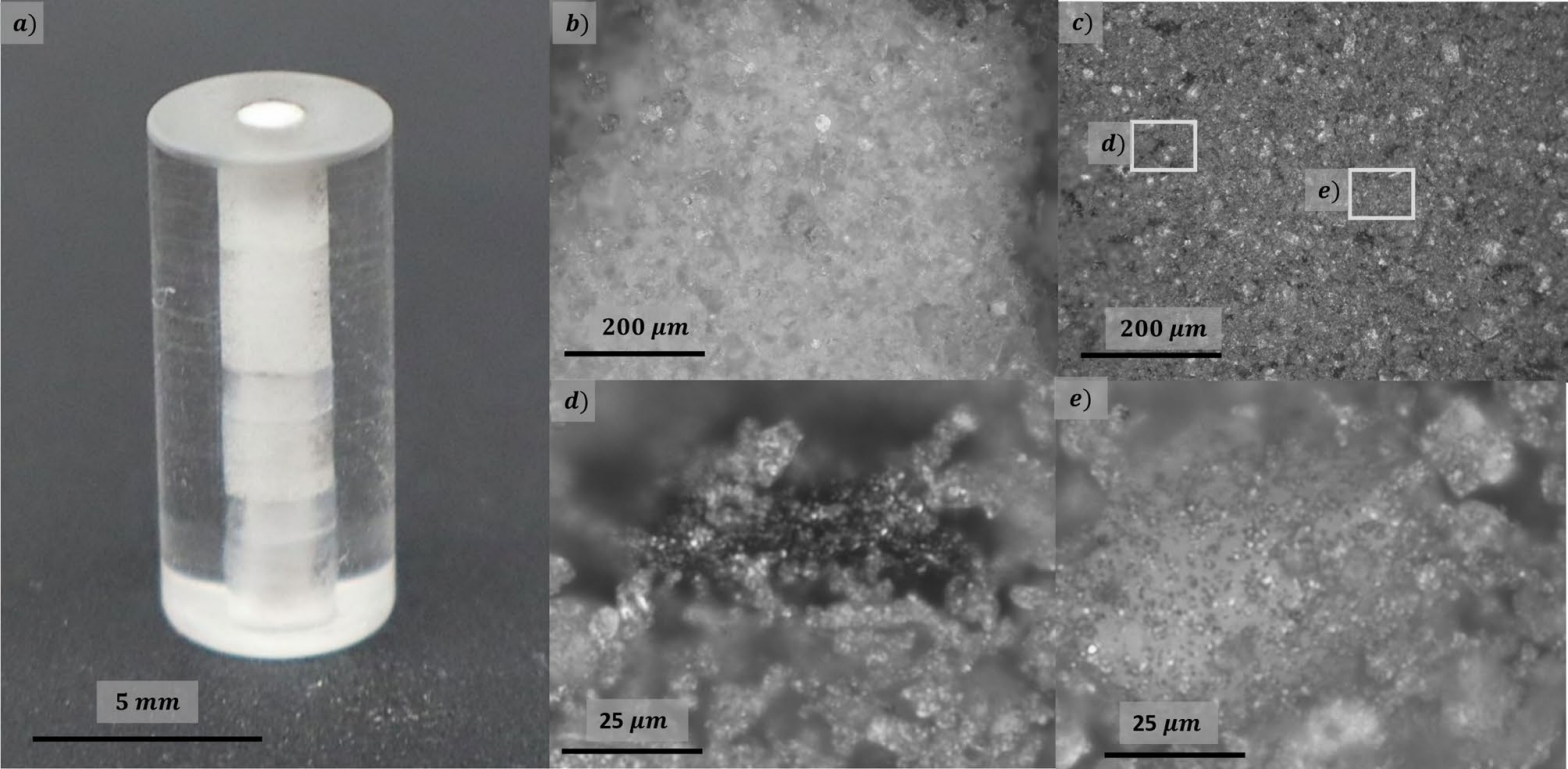
Sample overview and microstructural details. Left, (**a**) picture of a sucrose sample without the confinement medium. Right, microscopic images of (**b**) sucrose sample, (**c**) sucrose and aluminum nanoparticles mixture. (**d**, **e**) Zoomed microscopic images of different zones on sucrose and aluminum nanoparticles mixtures. They show that some nanoparticles form micrometric black clusters as in (**d**), yet generally exhibiting a homogeneous repartition on sucrose grains as in (**e**).

The average grain size distribution of the sucrose is centered on 34 μm, but the granulometry presents a relatively wide dispersion, ranging from 1.5 μm to 200 μm. Similarly to what is done with high explosives, the sucrose is pressed into a 1.5 mm diameter, 12 mm long PMMA cylinder tube (see Fig. 1a). The compression is performed in four stages, each compressing a 3-mm layer of material under a pressure of 700 bars. To withstand the high pressure during compression, the tube must be highly resistant, yet as thin as possible and transparent to X-rays. PMMA has very low linear absorption coefficient, below 0.2$\:\:\mathrm{c}{\mathrm{m}}^{-1}$ in the 19–35 keV X-ray domain^28^ and constitutes therefore an ideal material for this purpose. Once compressed, the sample is made of 80% sucrose and 20% air in weight. The mass fractions of air and sucrose in the sample are determined based on the mass of the pellet and the volume of the tube.

Aluminum nanoparticles were incorporated in some samples to favor the absorption of the laser and hopefully generate a stronger shock^29^. Aluminum nanoparticles being pyrophoric^30^, 250-nm oxidized aluminum nanoparticles are used. The mixing of the sucrose and 3% in weight of aluminum nanoparticles was performed using a Retsch agitator at 18 Hz for 20 minutes. The nanoparticles and the mock material are placed in a mixing container with a rubber ball, which serves to homogenize the mixture while preserving the integrity of the grains. Figure 1b and c present images on a microscope of the sucrose and sucrose with aluminum samples, respectively. Due to their nanometric size, some of the oxidized aluminum particles tend to aggregate in clusters ranging from 10 to 25 μm in size, as shown in zoomed microscopic images of sucrose and aluminum mixtures in Fig. 1d, but the repartition is generally rather homogeneous on the surface of sucrose grains (Fig. 1e). After compression, the doped samples are made of 83% in weight of sucrose, 0.02% oxidized aluminum nanoparticles, and 17% air. The resulting densities of the samples with and without aluminum can be found in Table 2.

### Nanosecond laser-induced shocks

For the laser-induced shock generation, the second harmonic ($\:\lambda\:=532\:\mathrm{n}\mathrm{m})$ of a Quantel Brillant B laser, delivering laser pulses of 5.3 ns duration and 350 mJ of energy on a single shot is focused through the confinement layer (acrylate-based polymer tape with a thickness of 2.5 mm), onto the upper surface of the material. The laser energy is adjustable and allows the control of the laser intensity. To determine the laser spot size, an aluminum adhesive tape is applied to the sample surface, and its ablation is measured, resulting on a spot diameter of 1 mm. Consequently, the average laser intensity at the surface of the material is calculated to be 8.4 GW/cm².

### Time-resolved X-ray imaging

**Fig. 2:**
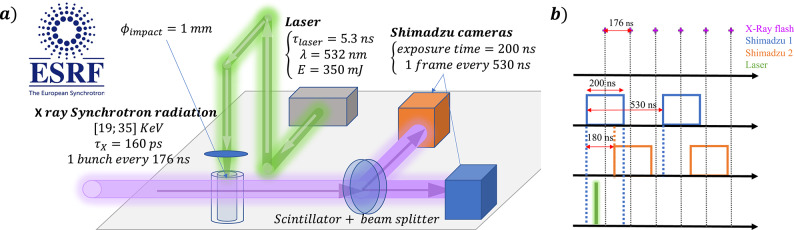
Time-resolved imaging setup and synchronization scheme. (**a**) 3D scheme of the experimental setup. (**b**) Synchronization of the camera, laser pulse and X-ray flash.

Figure 2a illustrates the experimental setup^31,32^. The X-ray pulse passes through the sample, and the transmitted light is then converted into visible light by a scintillator. The visible light is split by a beam splitter and captured by two ultra-fast Shimadzu HPVX-2 cameras to obtain high temporal resolution. The ID19 beamline, where this experiment was conducted, is particularly suited for in-situ radiography^20^, as it is equipped with ultrafast cameras capable of capturing the dynamic responses of materials to shock waves. The synchrotron radiation provides polychromatic X-ray source suitable for high temporal resolution dynamic imaging inside materials, as it is both extremely bright and fast, with a short pulse duration of 0.16 ns every 176 ns, and an energy of 19–35 keV.

The frame rate of each camera is set to one-third of the repetition rate of the synchrotron. Each camera has a minimum exposure time of 200 ns and is set to capture one image every 530 ns. The cameras are synchronized with the X-ray pulses (Fig. 2b) so that the first X-ray pulse coincides with the exposure window of the first camera. Only one X-ray flash is used in each illumination sequence. The second pulse occurs 176 ns later, falling within the exposure window of the second camera. The third pulse is not captured because of the frame-rate limitation. The cycle then repeats. Two zoom configurations were available in this experiment: the first one (1x) corresponds to a field of view of 12.8 × 8 mm² and could be used on both cameras. The second one (4x) enabled imaging of the sample with a resolution four times higher but could only be used on one camera due to space constraints. This 4x zoom configuration has a field of view of 3.2 × 2 mm². The 1x configuration is therefore used for temporal resolution while the 4x configuration provides higher spatial resolution.

## Results

### Dynamic X-ray imaging of nanosecond laser-irradiated sucrose

**Fig. 3:**
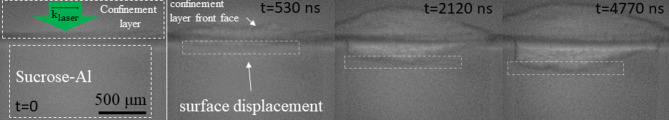
X-ray images of a laser-irradiated, aluminum-doped sucrose sample confined with an acrylate adhesive. Following laser impact, the individual surfaces move in opposite directions.

The initial stage of the experiment, captured just before any motion of the interface occurs, shows the sample at rest with an acrylate layer taped at the top of the assembly (see Fig. 3 at t = 0). During a laser shock, the effects can be amplified if the generated plasma is confined^33^. In that case, the plasma expansion is constrained by a confinement medium, and the shock wave pressure reached is higher and lasts longer^34^. Therefore, we chose to tape a layer of acrylate adhesive, transparent in the visible range, to the laser-illuminated surface of the samples^34^.

The laser beam is incident from above with a spot size several times larger than the average grain size. The sucrose sample is enclosed within the PMMA tube. This configuration, recorded using the 4x zoom setting of the imaging system, establishes the baseline for observing the response of the material to the laser-induced plasma generation. After the laser energy is deposited and plasma formation occurs, the sucrose experiences high pressure. Figure 3 illustrates the hydrodynamic response of the material to the pressure wave, as evidenced by the displacement of the upper face of the material. The deformation of the acrylate confinement layer (transparent in the visible range) shows that the plasma generated by the laser creates a pressure wave propagating in all directions. The sucrose surface motion is quantified and further analyzed.

### Particle motion under dynamic loading

In doped samples, aluminum grain clusters appear darker in the images of aluminum and sucrose mixture samples due to their higher density relative to air and sucrose. This results in increased X-ray absorption and creates a high contrast with the surrounding material. These clusters range in size from 10 to 25 μm, smaller than the 30 μm resolution of the 1x zoom configuration but larger than the 8 μm resolution of the 4x zoom configuration. The high spatial resolution of the 4x zoom configuration allows for the identification and tracking of these aluminum grain clusters within the sample. In the 4x zoom experiments, it was found that the local displacement of the interface at the center of the laser impact corresponds to the displacement of an aluminum cluster within the sample, along the same axis. As such, the interface velocity measured with high temporal resolution in the 1x zoom configuration can be approximated as the particle velocity. The position of the interface is tracked using image analysis, and image processing techniques are applied to enhance contrast and better highlight the interface.

**Fig. 4:**
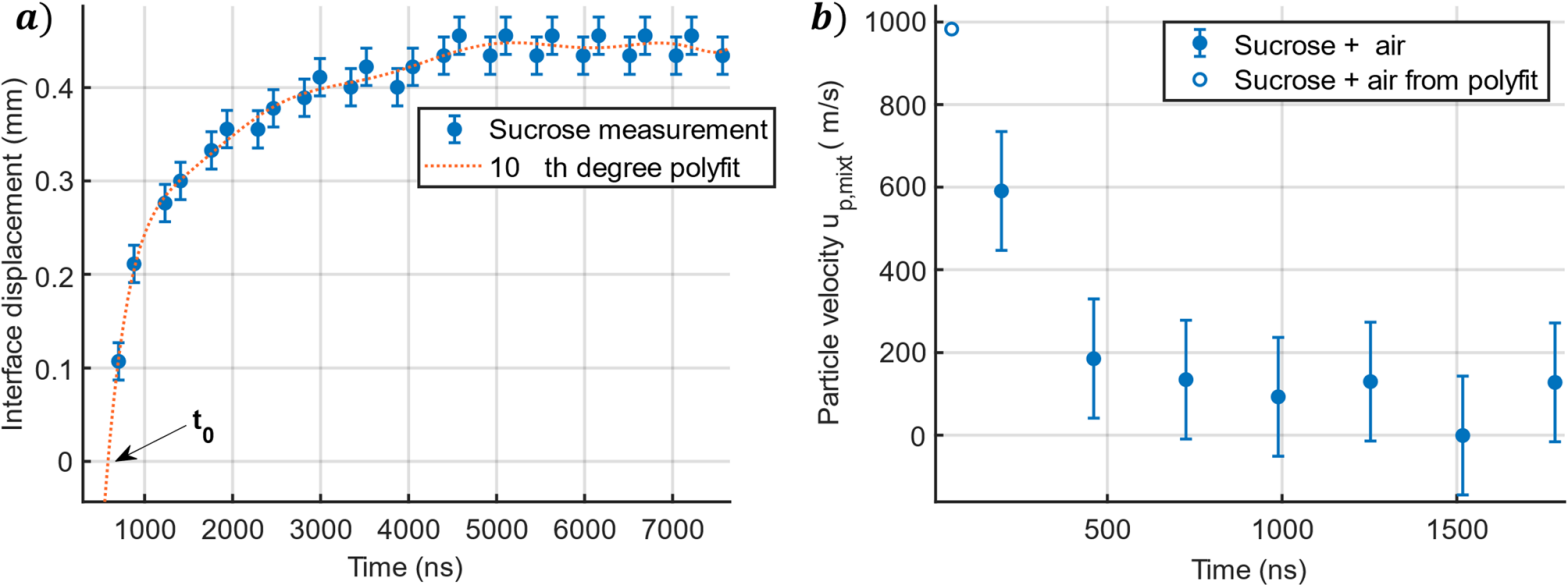
Particle dynamics measurements. (**a**) Interface displacement over time for a sucrose sample and (**b**) particle velocity over time for a sucrose sample.

The precise timing of the laser energy deposition could not be determined due to limited timing diagnostics available during the experiment. However, we systematically observed a flash of light in the first dynamic image, that we suppose originates from the laser, as no other pulsed light source was in the field of view of the cameras. Since the exposure time of the camera is of 200 ns, this means that the laser occurs at the earliest 200 ns before the X-ray flash on the first dynamic picture. A good approximation of the time of laser energy deposition, denoted $\:{t}_{0}$, is obtained by applying a 10^th^ order polynomial fit to the displacement data. In this case, $\:{t}_{0}$ is defined as the time for which the displacement starts. For the sucrose sample analyzed in Fig. 4a, the fit on the displacement reaches the horizontal axis at an estimated $\:{t}_{0}$, which is 109 ns before the first measured displacement. This approach provides consistent $\:{t}_{0}$ values for all samples and within the defined time interval. Figure 4a shows the interface displacement over time for a sucrose sample. The first measured displacement occurs 109 ns after the estimated energy deposition. The error on the interface displacement measurement is 38 μm and corresponds to the uncertainty caused both by the spatial resolution of the image in the 1x zoom configuration, and by the slight difference in the field of view of the two cameras. The data points derived from the interpolations are not subjected to error as the interpolation provides a precise value of $\:{t}_{0}$. However, those points should be treated cautiously as a reasonable estimation and not a measurement. The estimated $\:{t}_{0}$ is used as the time reference for the analysis.

Particle velocity - shown in Fig. 4b for a sucrose sample, over time—is defined as the time derivative of the interface displacement and corresponds to the average velocity of the material between two measurement points. As an example, the time averaged velocity between the first and second measured data points, corresponds to 590 $\:\pm\:$ 143 m/s.

Nanosecond laser-induced perturbations in confined regime typically last several hundreds of nanoseconds^34^. Therefore, this value might not be the maximum velocity reached as it is calculated between 109 ns and 637 ns after the energy deposition. Assuming the velocity is equal to 0 at $\:{t}_{0}$, the average particle velocity during the first hundred nanoseconds reaches 982 m/s. The high particle velocity is strong evidence that a pressure wave has been generated, and propagates through the material, causing a high-velocity matter displacement along the wave propagation axis. To pursue with further analysis, we determine whether this pressure wave is a sonic compression wave, or a shock wave.

### Assessment of shock generation in nanosecond laser–irradiated sucrose using *post mortem* X-ray tomography

**Fig. 5:**
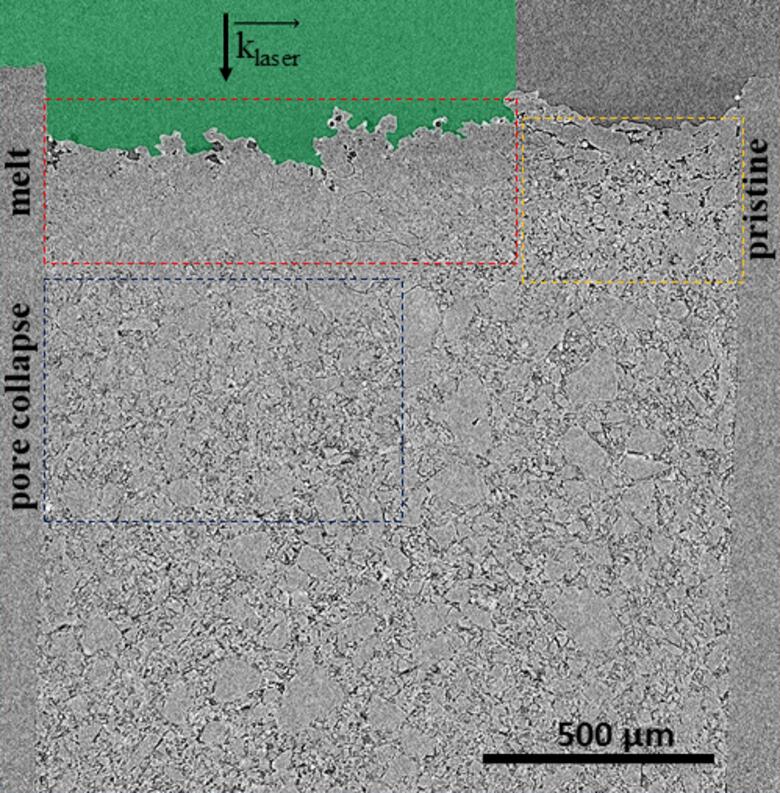
X-ray tomographic analysis of a shocked sample. The laser spot is position is added for reference. Below the laser-irradiated zone, a melted area is visible (red rectangle) as well as a pore collapse zone (blue rectangle). A non-irradiated zone is highlighted for reference (yellow rectangle).

In order to identify the nature of the generated pressure wave, we employed high-resolution X-ray tomography to investigate the internal microstructure of both a laser-illuminated sample. This approach allows for detailed examination of the microstructure, such as the porosity and the grains sizes and shapes, without altering or damaging the sample. The microtomography was performed on the upper part of the sucrose samples (on the face of the laser impact). The energy was 80 keV, allowing us to achieve very high resolution of 1 μm/voxel. Typical results are presented in Fig. 5. Compared to the non-irradiated part of the sample, the laser impact shows a melted region in the first few hundred micrometers (red rectangle in Fig. 5). The pores appear noticeably smaller beneath the melted region, and the grains are fragmented, with some broken and others bonded together (blue rectangle in Fig. 5), compared to a zone farther from the impact area within the same sample (yellow rectangle in Fig. 5). Additionally, jetting has been observed in the dynamic data (not shown here), which typically occurs after pore closure, indicating that the material has reached a fully compressed state^35^, and results from the surplus energy remaining after pore collapse.

These observations are consistent with a dynamic compaction regime as described by Ghosh et al.^12^, where excess energy after pore collapse leads to localized melting, and jetting. In the quasi-static regime, however, all the energy is consumed in pore collapse without melting or jetting. The clear signs of melting, pore size reduction, and jetting thus provide strong evidence that the wave propagating through the material was not a mere elastic or plastic compression wave traveling at the sound speed, but a true shock wave characterized by high-pressure loading above the critical threshold necessary to launch it. Knowing the nature of the wave allows the use of the shock wave equations to quantitatively characterize its pressure and velocity.

### Determination of the shock wave velocity and pressure

From the measured particle velocity together with the shock parameters of the constituent materials, one can determine the shock velocity and the produced pressure. Using Torvik’s mixing model^36^, the inverse of the initial (pre-shock) density of a mixture is written as $\:\frac{1}{{\rho\:}_{0,mixt}}={\sum\:}_{i}\frac{{f}_{m,i}}{{\rho\:}_{0,i}}$, where $\:{f}_{m,i}\:$is the mass fraction and $\:{\rho\:}_{0,i}$​ the initial density of component i (e.g., air, sucrose, and, if present, aluminum). After passage of a shock of pressure P, the mixture density $\:{\rho\:}_{mixt}\left(P\right)$ follows $\:\frac{1}{{\rho\:}_{mixt}}={\sum\:}_{j}\frac{{f}_{m,j}\:}{{\rho\:}_{0,j}}{\left(\frac{P}{{A}_{\mathrm{j}}}+1\right)}^{-\frac{1}{{n}_{\mathrm{j}}}}+\frac{{f}_{{m}_{0},air}}{6{\rho\:}_{0,air}}$, air being considered as a perfect gas with γ = 1.4. The material parameters are $\:{n}_{j}=4{s}_{j}-1$ and $\:{A}_{j}=\frac{{\rho\:}_{0,j}{c}_{0,j}^{2}}{{n}_{j}}$​​, where $\:{s}_{j}$, $\:{c}_{0,j}$ (bulk sound velocity) and $\:{\rho\:}_{0,j}$​ are tabulated constants for component j being either sucrose or if applicable aluminum. These equations remain valid in strongly porous mixtures for shock pressures below 25 GPa, a hypothesis that will be validated later on.

From those equations, the particle velocity $\:{u}_{p,mixt}\left(P\right)$ and shock velocity $\:{u}_{s,mixt}\left(P\right)$ as a function of the pressure P on the mixture are computed using Torvik’s mixing model, derived from the Rankine-Hugoniot Eq.^37^:

1 $$\:{u}_{s,mixt}\left(P\right)=\sqrt{\frac{P}{{\rho\:}_{0,mixt}\left(1-\frac{{\rho\:}_{0,mixt}}{{\rho\:}_{mixt}\left(P\right)}\right)}}$$

2 $$\:{u}_{p,mixt}\left(P\right)=\frac{P}{{\rho\:}_{0,mixt}.{u}_{s,mixt}}$$

Plotting $\:{u}_{s}=f\left({u}_{p}\right)$ allows to retrieve the sound velocity $\:{c}_{0,mixt}$ and the shock parameter of the mixture $\:{s}_{mixt}$ using the empirical formula^38^:

3 $$\:{u}_{s,mixt}={c}_{0,mixt}+{s}_{mixt}.{u}_{p,mixt}$$

This formula remains an approximation for non-metallic materials but provides a good estimation of the shock velocity. Table 2 provides the density, speed of sound and shock parameter for raw materials, and for the compressed mixture of air-sucrose and air-sucrose-aluminum.

**Table 2:** Density, sound speed and shock parameter of sucrose, aluminum, compressed mixture of sucrose and air, and compressed mixture of sucrose, air, and aluminum.

Material	Density (g/cm^3^)	$\:{c}_{0}$ (m/s)	s
Sucrose^27^	1.58	3040	2.05
Aluminum^39^	2.78	5370	1.29
Sucrose-air compressed mixture	1.19	743	2.36
Sucrose-air-aluminum compressed mixture	1.25	865	2.47

**Fig. 6:**
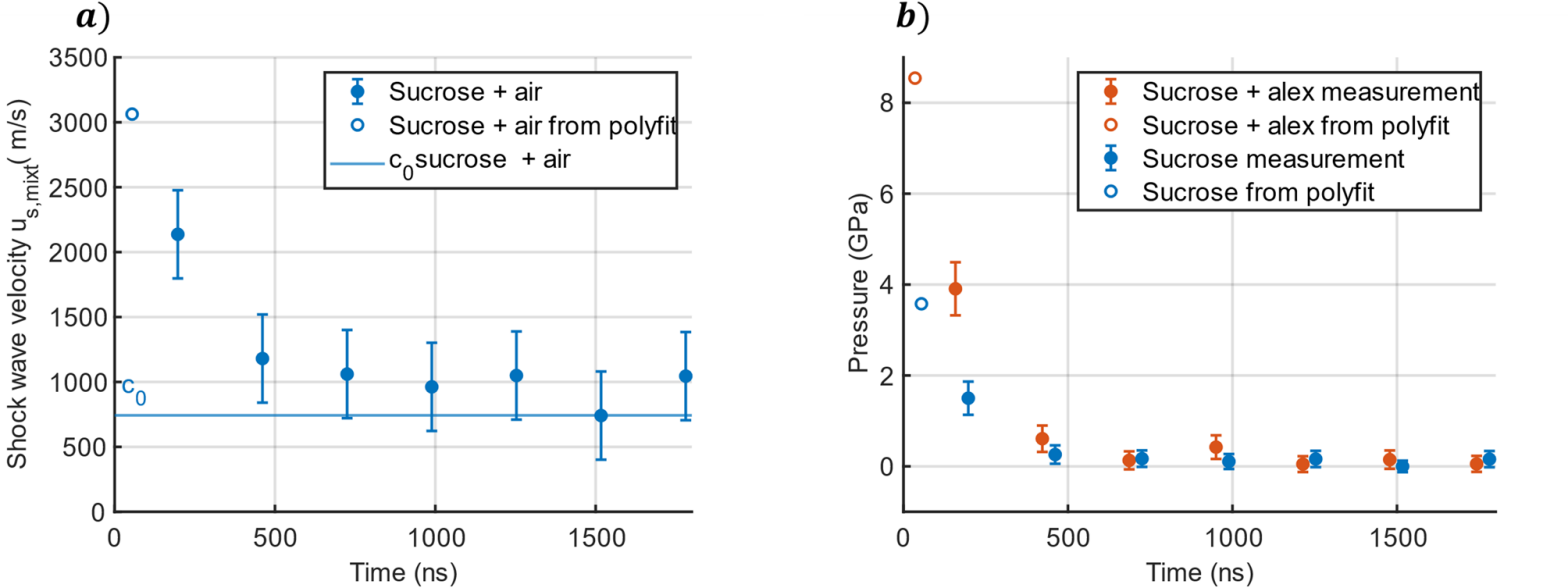
Shock wave characteristics. (**a**) Velocity over time for a sucrose sample. (**b**) Shock pressure over time for a sucrose sample (blue) and a sucrose-oxidized aluminum nanoparticles mixture (orange, denoted “sucrose+alex”).

The shock wave velocity is calculated using the Eq. (3) and the mixture parameters, and presented in Fig. 6a for the studied sucrose sample. The average shock wave velocity on a sucrose sample, calculated during the first 109 ns, assuming that the interface displacement is null at $\:{t}_{0}$, is 3062 m/s – which is more than four times higher than the sound velocity of the material, 743 m/s. The average shock velocity calculated between 109 ns and 637 ns from measured-only interface displacement, is 2137 ± 339 m/s. At the low end of the error range, this velocity is well above the sound velocity in the material, which is characteristic of a shock wave.

In order to determine whether the generated shock is strong enough to initiate a shock-to-detonation transition in a high explosive, a key indicator is the built-up pressure in the mock explosive. Since negligible amounts of matter is ablated during a laser shock^10^, we can directly apply Eq. (2) to extract the resulting pressure in our experiment. Considering the extrapolated $\:{t}_{0}$, the peak pressure value was estimated around 3.6 GPa, as shown in Fig. 6b. The calculated pressure at the first measurement point for the sucrose sample was 1.5 ± 0.4 GPa. Similar analysis on compressed sucrose samples containing aluminium nanoparticles has been conducted. The beginning of the laser shock was estimated to occur 70 ns before the first dynamic image. Figure 6b presents a comparison of the pressure levels for the samples without and with aluminum nanoparticles, showing that higher pressure levels are reached in the second case. The highest estimated pressure in the sample with aluminum was 8.5 GPa. In all cases, the extracted pressure values justify the use of Torvik’s mixing model as they remain well below 25 GPa.

## Discussion

Using time-resolved X-ray imaging on nanosecond laser–irradiated mock-explosive samples, we observed and quantified the generated wave properties. We directly measured a shock with peak pressure of ~ 3.6 GPa and an average velocity corresponding to ~Mach 4 (see Table 2 for sound speed at rest) in a granular, low-absorption material (compressed sucrose). Doping the sample with aluminium nanoparticles, which increase laser absorption, raised the peak pressure to ~ 8.5 GPa. These pressures are consistent with previously reported values for confined nanosecond laser shocks^40^.

A central question motivating this study was whether the generated wave is sufficient to initiate the transition of an explosive to detonation. Although a peak pressure of this magnitude can initiate some high explosives when applied over millisecond timescales^41^, the considerably shorter duration of laser-driven shocks (typically a few hundred nanoseconds) and the rapid pressure decay make initiation less likely in this case. In comparable experiments, initiation of energetic materials such as HNS typically requires ~ 10 GPa applied for ~ 10 ns, or ~ 5 GPa for shocks lasting ~ 100 ns^42^. The dependence of the initiation threshold on the laser spot size has also been highlighted, in particular with respect to the critical diameter of the explosive^43^. Our results suggest that mixing the energetic material with a small fraction of absorbing particles could be an effective strategy to increase laser absorption and reach the pressure levels needed to initiate detonation via a laser-generated shock.

## Conclusions

In this report, we demonstrate the generation of a shock wave in a compressed sucrose-based mixture using nanosecond green laser pulses. By employing high-speed, single-bunch X-ray imaging at a bright synchrotron source, we directly observe the particle dynamics induced by the laser-mixture interaction. Ex situ X-ray microtomography further confirms the generation and propagation of the shock wave. Using a Hugoniot-based framework, we estimate a peak shock wave pressure of approximately 8.5 GPa.

Given that the mixture mimics the behaviour of common high explosives, our findings provide a foundation for developing rapid-response opto-pyrotechnic detonators. In future experiments, measuring the shock wave duration will enable a comprehensive evaluation of shock wave-induced detonation in real explosives.

## Data Availability

Data underlying the results presented in this paper are not publicly available at this time but may be obtained from the authors uponreasonable request.
